# Exploring the links between cancer and placenta development

**DOI:** 10.1098/rsob.180081

**Published:** 2018-06-27

**Authors:** Vincenzo Costanzo, Alberto Bardelli, Salvatore Siena, Sergio Abrignani

**Affiliations:** 1 IFOM, The FIRC Institute of Molecular Oncology, University of Milan Medical School, Milan, Italy; 2 Department of Oncology, University of Milan Medical School, Milan, Italy; 3 Candiolo Cancer Institute-FPO, IRCCS, University of Turin, Candiolo, Turin, Italy; 4 Department of Oncology, University of Turin, Candiolo, Turin, Italy; 5 Niguarda Cancer Center, Grande Ospedale Metropolitano Niguarda, Milan, Italy; 6 INGM, Istituto Nazionale Genetica Molecolare “Romeo ed Enrica Invernizzi”, Milan, Italy; 7 University of Milan Medical School, Milan, Italy

**Keywords:** DNA repair, DNA damage response, cancer

## Abstract

The development of metastatic cancer is a multistage process, which often requires decades to complete. Impairments in DNA damage control and DNA repair in cancer cell precursors generate genetically heterogeneous cell populations. However, despite heterogeneity most solid cancers have stereotypical behaviours, including invasiveness and suppression of immune responses that can be unleashed with immunotherapy targeting lymphocyte checkpoints. The mechanisms leading to the acquisition of stereotypical properties remain poorly understood. Reactivation of embryonic development processes in cells with unstable genomes might contribute to tumour expansion and metastasis formation. However, it is unclear whether these events are linked to immune response modulation. Tumours and embryos have non-self-components and need to avoid immune responses in their microenvironment. In mammalian embryos, neo-antigens are of paternal origin, while in tumour cells DNA mismatch repair and replication defects generate them. Inactivation of the maternal immune response towards the embryo, which occurs at the placental–maternal interface, is key to ensuring embryonic development. This regulation is accomplished by the trophoblast, which mimics several malignant cell features, including the ability to invade normal tissues and to avoid host immune responses, often adopting the same cancer immunoediting strategies. A better understanding as to whether and how genotoxic stress promotes cancer development through reactivation of programmes occurring during early stages of mammalian placentation could help to clarify resistance to drugs targeting immune checkpoint and DNA damage responses and to develop new therapeutic strategies to eradicate cancer.

## 1. Introduction

Cancer is a multistage disease that affects millions of people on this planet. Development and progression of cancer can be driven by the acquisition of genome instability, which is facilitated by stressful conditions affecting the DNA replication process, including high proliferation rate, low DNA repair capacity and exogenous or endogenous insults to DNA. The acquisition of an unstable genome predisposes to the emergence of genetically distinct sub-clonal cell populations and intra-tumour heterogeneity, which pose major challenges in understanding cancer, managing patients and designing effective treatment strategies [1–3]. However, although heterogeneous, most solid cancers have stereotypical behaviours that involve phases of growth, expansion, stabilization and acquisition of malignant properties such as tissue invasiveness, immune evasion and stimulation of angiogenesis. The heterogeneous nature of cancer cells is difficult to reconcile with the occurrence of these common behaviours. Tumours heavily rely on adaptive responses to DNA metabolism impairments for their continued proliferation as in the case of replication stress (RS), which can be defined as the presence of multiple alterations affecting DNA replication intermediates [4], and replication stress response (RSR) [5–8]. As cancer cells recapitulate several aspects of embryogenesis, including rapid proliferation and consequent RS, they could also hijack the suppression mechanisms that embryos put in place against the maternal immune response towards fetal neo-antigens. These mechanisms are extremely powerful at repressing the maternal immune response and rely on a large number of molecules and pathways, some of which are targets of cancer immunotherapy, such as PD-L1 [9]. These processes are orchestrated by the trophoblast, which is made of unique cell types that evolved recently in mammalian organisms [10]. The trophoblast forms the outer layer of the blastocyst. Its function is to provide nutrients and shelter to the embryo through the formation of the outer chorionic sac and the fetal portion of the placenta. Trophoblast cells are unique and evolved by co-opting genes normally expressed elsewhere in the organism. During this process, few completely novel genes appeared in the genome of mammals, whereas others were derived by coevolution of duplicated genes and from horizontal transfer mostly due to retroviral insertions [11]. The trophoblast orchestrates the invasion of the endometrium and the attachment to the uterus wall, the building of new vessels connecting the maternal to the fetal circulation and the suppression of the maternal immune responses against fetal neo-antigens, making possible the bearing of live young in mammalian organisms.

Strikingly, cancer cells recapitulate many of these features. It is, therefore, possible that reactivation of trophoblast/placenta programmes in cancer cell precursors contributes to tumourigenesis. Most importantly, reactivation of trophoblast-specific pathways could contribute to the inactivation of lymphocyte-mediated control of tumour growth by repurposing pathways normally active during placenta formation and normally required to prevent maternal immune response against fetal antigens. This hypothesis posits that the so-called ‘pseudo-malignant’ trophoblast and cancer cells exploit comparable mechanisms at molecular level to achieve their proliferative, immunosuppressive and invasive processes [12–14]. A corollary to this hypothesis is that evolution of feto-maternal immune tolerance and invasive placentation might have also favoured the emergence of mechanisms for cancer metastasis in mammals, in which cancer occurs with high frequency [15]. Here, we explore the possible links between solid cancer development and mammalian placentation that could have contributed to the display of cancer features.

## 2. Genotoxic stress in early cancer precursors

A key feature of cancer cells is the presence of multiple signs of exposure to genotoxic stress resulting in widespread genome instability. The source of this stress might be ascribed to deregulation of normal DNA stability maintenance processes, in particular during DNA replication. DNA lesions and defects in the apparatus that carries out DNA replication, including lack of nucleotides, and promotes DNA repair can induce RS [16]. Significantly, RS can be elicited by oncogene activation [6] and lack of functional DNA repair proteins such as RAD51 and BRCA2 that operate at replication forks and protect nascent DNA from Mre11 nuclease-mediated degradation [16–19].

**Figure 1.**
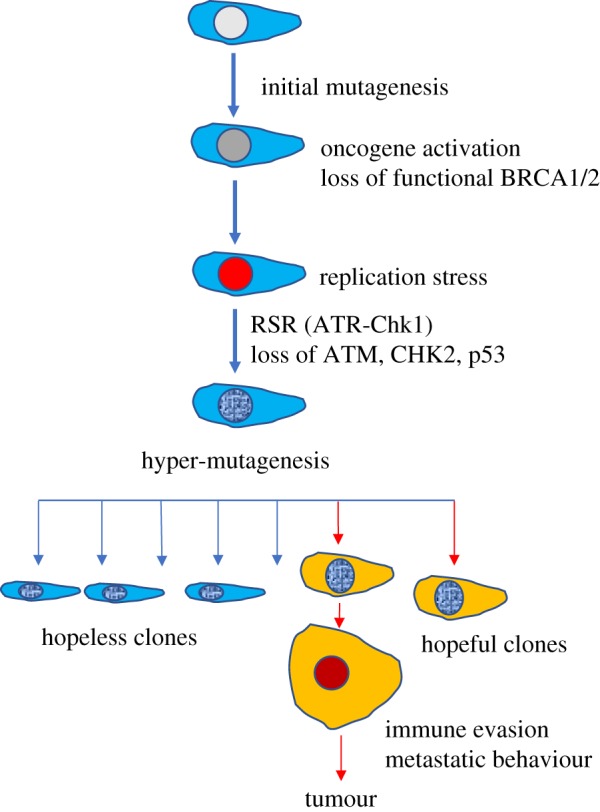
Emergence of cancer features by selection. Mutagenesis, oncogene activation, loss of functional BRCA1/2, accumulation of RS and loss of p53-mediated tumour barrier might predispose to hyper-mutagenesis-mediated emergence of clones that are positively selected for their ability to evade immune response and invade tissues.

The effects of RS have been studied in primary somatic cells, which respond to it by promoting cellular senescence through activation of the ATM–p53 axis [6]. Activation of this pathway has been shown to act as a barrier to tumour progression (hence referred to as *RS tumour barrier*) in early cancer lesions and adenomas [20,21] (figure 1). The ‘RS tumour barrier’ can be overcome following the loss of ATM, Chk2 or p53, often observed in tumour cells. RS is monitored by the ATR–Chk1 pathway, which is activated in the presence of extensive RPA-coated DNA and aberrant double- to single-stranded junctions in the context of double-strand breaks and stalled replication forks [4]. Activation of the ATR–Chk1-dependent response is observed in early cancer precursors indicating the presence of RS [6]. However, in contrast to ATM–p53, ATR–Chk1 loss is more rarely observed in cancer cells as these proteins are essential for cell survival. Consistent with this, decreased levels of ATR and treatment with ATR inhibitors (ATRi), presently being tested in phase I–II clinical trials, suppress tumour growth [22–25], whereas an extra copy of Chk1 facilitates cellular transformation [26].

The ATM–p53-dependent tumour barrier relies on the activation of the senescence programme and the acute elimination of damaged cells through apoptosis [6]. Although the loss of the RS tumour barrier is essential to progress from early lesions to the later stage of cancer development, it is not clear how this progression takes place.

Cancer features such as the ability to invade tissues and evade immune responses might emerge in a continuous stochastic evolutionary process that could be positively selected (figure 1). According to this, evolution of multiple cancer cell clones, as tracked by mutation analysis, can lead to the formation of cells with genetic changes predisposing to the acquisition of malignant features. These cells can remain silent or can be positively selected for their ability to grow and bypass immune responses, behaving as ‘hopeful monsters’ [27]. However, as cancer occurs with high frequency and develops fast once reaching a detectable size it is not clear whether the rate at which these clones evolve is sufficiently high to drive tumour formation. Also, it is unlikely that complex features such as the ability to invade tissues arise from a completely stochastic process. The driving force leading to the selection of an invasive behaviour remains largely unexplained, assuming that the tumour microenvironment operates a limited selection of evolving tumour cells. Finally, the high mutation frequency might negatively impact on the fitness of the emerging clones, favouring instead the formation of ‘hopeless monsters’, which are likely to die or remain silent [27] (figure 1).

**Figure 2.**
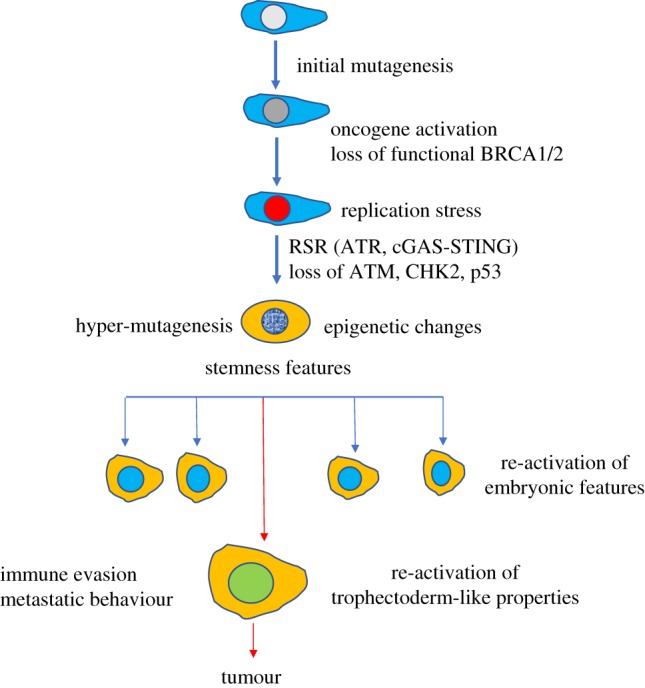
Emergence of cancer features by reactivation of embryonic pathways: Mutagenesis, oncogene activation, loss of functional DNA repair genes such as BRCA1/2, accumulation of RS, activation of RS-induced inflammatory pathways mediated by cGAS-STING and loss of ATM/p53-mediated tumour barrier might induce epigenetic changes predisposing to re-emergence of stemness and embryonic-like features alongside hyper-mutagenesis, which could boost this process. Full activation of trophectoderm-like properties might predispose to immune evasion and tissue invasion.

One possibility to explain the emergence of cancer clones with a partial or full spectrum of malignant features is the reactivation, possibly at a transcriptional level, of embryonic pathways, encoding for complex biological processes such as tissue invasion, cell migration and angiogenesis (figure 2). This state could be stably inherited by malignant clones, which could perpetuate the acquired properties in the presence of an evolving mutational spectrum supporting the development of malignant features. This hypothesis implies that early transformation events, likely triggered by RS, somehow impose an inheritable cell fate change acquired through cellular reprogramming and/or dedifferentiation to a status that recapitulates early embryonic development. These changes might be driven by DNA damage and DNA damage response and might occur in parallel with stochastic mutagenic events, which might positively affect cell fate changes. These events, when taking place in adult stem cells might not lead to senescence or cell death due to weaker checkpoint mechanisms, predisposing instead to the emergence of embryonic properties in cells that are less differentiated. The de-repression of endogenous programmes normally active during embryogenesis such as the epithelial to mesenchymal transition (EMT) [28] or the formation of extra-embryonic tissues, including the placenta, might be responsible, at least in part, for the acquisition of features such as the ability to evade immune control and invade surrounding tissues. These changes could be mediated by epigenetic reprogramming to more undifferentiated states. Establishment of epigenetic memory could then be responsible for the stable inheritance of these features.

Importantly, cell fate transitions might be favoured by the presence of an inflammatory state activated by RS and consequent chromosome instability [29]. In this case, chromosomal instability and/or loss of replication fork protection mechanisms induced by inactivation of fork protection genes frequently mutated in cancer such as BRCA1, BRCA2 or ATM might generate cytosolic DNA promoting the activation of the cGAS-STING cytosolic DNA-sensing pathway and downstream non-canonical NF-κB signalling [30]. These pathways are active in metastatic cells and might link genome instability to EMT and inflammation [29]. Intriguingly, cancer prone mutations in SAMHD1, which is required for replication fork stability and regulation of Mre11-dependent degradation of nascent DNA, activate the IFNγ pathway promoting cancer development [31]. Collectively, these and other evidence indicate a direct link between genotoxic stress response and activation of inflammatory pathways promoting cancer.

The impact of these pathways might be significant when occurring in stem cells, derailing their developmental programme. As it has been suggested that cancer frequency in different tissues correlates with the number of cell divisions of tissue-specific stem cells [32], it would be of great value to assess the effect of RS in stem cells and test whether RS and RSR impose a change in cell fate that recapitulates early embryonic programmes.

Alternatively, these same pathways might directly lead to the emergence of stem cell like properties in somatic cells [33–35], in line with results showing stem cell signalling and transcriptional pathways active in several tumours [36,37]. Notably, activation of TLR3-dependent innate immune responses, which are in part shared with the cGAS-STING pathway, is required for efficient cellular reprogramming [38]. Also, *in vivo* reprogramming drives Kras-induced cancer development [39]. Collectively, these findings indicate a strong link among the triad of inflammation, cellular reprogramming and cancer development. Interestingly, recent survey of chemoresistant triple negative breast cancers has highlighted the activation of convergent transcriptional programmes induced by neoadjuvant chemotherapeutic treatments based on taxane and anthracyclines. These programmes lead to the re-emergence of embryonic properties, including the ability to degrade the extracellular matrix, withstand hypoxia, undergo EMT and promote angiogenesis [40]. It would important to understand whether this is a common behaviour of cancer cells treated with agents inducing further genome instability and whether these signatures emerge also in response to DNA damage occurring at earlier stages of cellular transformation. These findings might also impact on our understanding of the effects of chemotherapy based on DNA damaging agents, which on the one hand kill proliferating cells, but on the other hand might contribute to activating pathways that are detrimental for residual cancer cells.

## 3. Immunological control of tumour development

Together with genome instability, a major hallmark of cancer cells is their ability to evade immune responses. Indeed, if successful tumour growth depends initially on genetic and epigenetic changes of tumour cells it then relies on the molecular editing these changes impose on immune cells, in particular T lymphocytes. Cancer cells must escape T lymphocyte responses to develop endless growing tumours. The interplay between tumour cells and the immune system is defined as cancer immunoediting [41]. The most complex forms of immunoediting are the adaptive responses mediated by CD4 and CD8 tumour-specific T lymphocytes driven by tumour neo-antigens, resulting in either tumour elimination, equilibrium between immune surveillance and tumour growth, or tumour escape from immune responses [41]. Furthermore, in parallel to the relations between neo-antigenic profile of tumour cells and tumour-specific T-cell responses, tumour proteins can affect T-cell functions in the tumour microenvironment, as is the case when tumour cells favour T regulatory (T-reg) suppressor cell recruitment at tumour sites, and effector T cells are rendered functionally inactive by T-reg cells even in the presence of very antigenic tumour cells [42]. In fact, once tumours become detectable with current diagnostic tools, namely when they reach millimetre size range, immune responses are no longer capable of effectively eliminating cancer cells. However, the recent successes of immunotherapy with checkpoint inhibitors demonstrated that, at least for highly antigenic tumours, it is possible, even in metastatic patients, to rescue T lymphocyte responses that can eliminate cancer cells and control or even eradicate tumours [43]. Among the tumours that respond to checkpoint inhibitors are the ones that display inactivation of the mismatch repair (MMR) system, which recognizes and corrects base mispairs, insertions and deletions that occur during DNA synthesis [44]. MMR-defective tumours represent approximately 20% of human tumours and have peculiar properties, which include early onset, metastatic potential but generally favourable prognosis, and remarkable response to immune checkpoint blockade. The biological and clinical features of MMR-deficient tumours are thought to be associated with their intrinsic ability to continuously generate new mutations, leading to increased levels of neo-antigens, which in turn trigger effective immune surveillance [45]. However, it is also conceivable that MMR deficiency (another form of genotoxic stress) might lead to transcriptional reprogramming leading to suppression of immune surveillance. Consistent with these observations, in an animal model of tumours with defective DNA repair, there is evidence that increasing mutational load (and thus the neo-antigen burden) in colorectal cancer by DNA-alkylating agents might sensitize to immune checkpoint blockade [46]. However, a number of tumours remain resistant to these treatments and the causes of this resistance are unknown [47]. While it is largely agreed that a high number of mutations (greater than 10 mutations/megabase) in cancer cells is required to obtain response to immunotherapy with checkpoint inhibitors, there is little explanation as to why the majority of patients with highly mutated tumours do not respond to this immunotherapy [47]. A deeper understanding of the immune responses elicited by cancer cells, also comparing escape mechanisms from immune responses in unmutated cells such as trophoblast cells could help detailing the mechanisms behind the immunosuppressive features displayed by the tumour environment.

## 4. Placentation: an embryonic process linked to cancer development in mammals

A number of cancer features can be recapitulated by an embryonic process unique to mammals, namely the formation of the placenta. Placentation is a complex multistage process leading to development of a disposable infrastructure that allows fast, efficient and regulated development of most mammalian organisms. This process starts with the contribution of extra-embryonic cells that form a vascularized adhesion plaque in the context of the maternal decidua, the modified region of the endometrium, the inner layer of the uterus, to which the embryo will adhere [48]. Many of the mechanisms leading to the formation of the placenta are still poorly understood [49].

**Figure 3.**
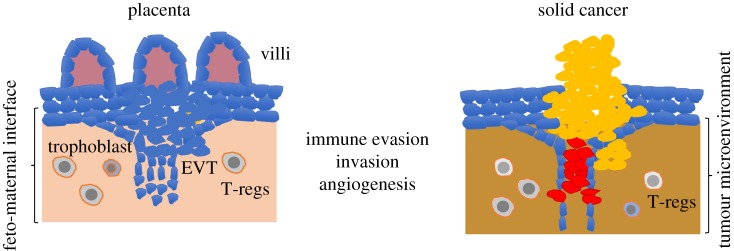
Parallels between placenta and solid tumour formation: solid cancer resembles placenta development in several aspects, including the formation of new vessels, and the ability to invade surrounding tissues and to evade the immune responses. Tumour microenvironment could be populated by cells similar to the ones found in the feto-maternal interface, where EVT invades the maternal tissues, including immunoregulatory T-reg cells.

Among the properties shared by trophoblast and cancer cells is the ability to invade healthy tissues, to form new vessels and to promote an environment that is protected from the immune system (figure 3). The rapid development of the embryo in placental mammals begins when the blastocyst attaches to the uterine wall [49]. This is a complex event that is orchestrated by the trophoblast at the outer layer of the blastocyst rapidly proliferating and invading the maternal decidua, thus leading to the formation of a mature placenta, which has an embryonic side and a maternal component. Notably, a recent analysis has shown that several genes that cause embryonic lethality when deleted have a primary function in placenta trophoblast cells [50], indicating the essential role of placenta for development.

There are three major types of placenta that can be found in different species. They are classified based on their degree of attachment and invasiveness. Epitheliochorial placentas are the least invasive, as they have three layers of maternal tissue separating the fetus from the maternal blood. Endotheliochorial placentas are instead partially invasive as only the endothelial wall of the maternal blood vessels and connective tissue separate the fetus from the maternal blood. Haemochorial placentas, which are the most diffuse type, are also the most invasive ones. In this type of placentas, fetal tissues are directly in contact with the maternal blood [51].

The embryonic side of the placenta comprises a number of anatomical structures that include the villi, which attach to the uterus. The villi in haemochorial placentas are made by columns of different cells, including cytotrophoblast cells, which constitute the major cell type of the placenta [48]. Some of these cells are markedly polyploid with large nuclei, similar to aggressive cancer cells. Cytotrophoblast differentiates into distinct cell types: the syncytiotrophoblast cells, forming the external layer of the villi, which are cells are terminally differentiated and are important for feto-maternal nutrient exchanges; and at the tip of the villus there are instead cells of the extravillous trophoblast (EVT), which have properties astonishingly similar to metastatic cancer cells. Among these, there is the ability to migrate into the uterus wall and to invade the uterus glands and vessels [52]. Following the invasion of the uterus wall EVT cells penetrate the maternal arteries substituting their endothelium. Similar to cancer cells EVT cells undergo EMT and secrete exosomes to prepare for tissue invasion [53]. In contrast to cancer-invading cells, EVT cells are eliminated at the end of pregnancy. The study of EVT cells might be useful to understand how cancer cells develop their invasive potential [53].

Owing to these invasion mechanisms that impact on the integrity of the receiving tissue, the process is markedly inflammatory. Inflammatory pathways are essential for a successful implantation as shown by the requirement for molecules such as prostaglandin E and inflammatory cytokines TNF and IL6 [10]. However, at some point, the placenta activates anti-inflammatory mechanisms necessary to support the continuation of the pregnancy by shielding the fetus from maternal immune-mediated attack [10]. Without these mechanisms, the presence of paternal genetic material acting as neo-antigens would trigger an immune-mediated attack against the fetus, which would be destroyed by the maternal immune system, terminating the pregnancy [54]. The fetus escapes rejection from the maternal immunity thanks to a multitude of immunomodulatory properties of the feto-maternal interface that allow the survival of the immunologically distinct fetus by imposing a strong feto-maternal tolerance [54,55]. Strikingly, some of the mechanisms driving feto-maternal tolerance are reactivated in cancer, raising the possibility of a straightforward parallelism between cancer predisposing elements and feto-maternal tolerance [56]. Consistent with this, recently, a number of placenta-related genes have been found to be highly expressed in metastatic lung cancer [57]. These tumours show increased expression of genes encoding nuclear factors promoting cell proliferation while downregulating genes involved in the control of the immune response.

## 5. Molecular pathways shared by placenta and cancer cells

There are several pathways shared between placenta and cancer cells at molecular level. These pathways regulate hyperproliferation, invasion, angiogenesis and immunoevasion. Similar to cancer cells, proliferation is supported by high levels of IGF/MAPK, activation of anti-apoptotic pathways based on BCL2 expression, multiple genome duplication events leading to polyploidy and several others. Sustained angiogenesis is instead promoted by the activation of VEGF, HIF1α and FGF-based pathways [9].

The invasive portion of the placenta is made up of the EVT. Several parallels can be made between invasive EVT cells and cancer cells [9]. Both cancer cells and trophoblast cells promote migration through activation of EMT, which leads to loss of cell-to-cell contact inhibition. Important for this process is the WNT pathway, the expression of proteins degrading the extracellular matrix and the change in integrin patterns favouring cell movements through tissues. A number of pro-metastatic genes have been shown to be overexpressed in cancer cells. Among these, there are integrin a7b1, TGF-β and VEGF. These genes appear to be regulated by HIF1α [58] and seem to play a role in placenta formation [9]. Also, HIF1α has been shown to support trophoblast differentiation [59], which in the early stages of embryo development takes place in hypoxic conditions due to lack of blood vessels.

EVT and more generally syncytiotrophoblast cells are markedly polyploid [9] and, similar to cancer cells, polyploidy might make trophoblast cells resistant to DNA damaging agents. Alternatively, resistance to DNA damage might also influence polyploidy occurrence, which might develop in response to DNA damage and RS. Consistent with this, cells derived from mice carrying mutations in Fan1 nuclease, which is involved in repairing DNA cross-links, undergo polyploidization in response to DNA cross-linking [60]. A similar situation might occur in placenta cells, which might develop polyploidy in response to persistent RS due to high levels of proliferation.

Modulation of the maternal immune system is a major challenge for the developing pregnancy. This process appears to be regulated in trophoblast cells by reduced expression of HLA class I cell surface proteins, expression of non-classical HLA class G with immunosuppressive properties [61], activation of enzymes that restrict the supply of tryptophan to immune cells such as indoleamine 2,3-dioxygenase (IDO) [62] and high expression of PD-L1 [63]. Furthermore, STING protein, which participates in IFNγ activation in response to foreign and self-DNA, has been shown to be highly expressed in placenta [64].

The placenta is also infiltrated by regulatory and immunosuppressive cells such as T-reg, which express CD25 on their surface [65]. The development of immune tolerance, which is critical to sustain placentation and live bearing, is indeed critically dependent on T-reg-based mechanisms, similar to cancer immunoediting processes [66]. Among the genes regulating T-reg cell specification, there is Fox3p, the expression of which requires the presence of CNS1 promoter element. The deletion of CNS1 leads to a specific downregulation of peripheral T-regs, inducing increased resorption of the semiallogeneic fetuses, a phenomenon that is not seen with syngeneic fetuses [65]. Notably, CNS1 is only present in placental mammals and likely evolved due to a transposon insertion in the Fox3p gene promoter locus. This observation suggests that evolution of T-reg cell based feto-maternal tolerance mechanisms played an essential role in the evolution of placentation. Finally, placental cells also produce exosomes containing immunosuppressive molecules such as PD-L1, similar to cancer cells [67,68].

These elements strongly support the links among cancer, placentation and development of immune tolerance. All these mechanisms are clearly shared with cancer cells, which activate pathways to invade tissues and escape immune control using the same molecules activated during placentation.

Similarities are not limited to cell behaviour and functional aspects but are present also at genome level. Placentation is indeed associated with widespread hypomethylation of CpG islands in trophoblast cells. Remarkably, global methylation status of cytosine in placenta cells is markedly similar to cancer cells [69,70]. Strikingly, this similarity is not limited to quantitative levels of methyl cytosine but involves the presence of similar patterns of hyper- and hypomethylation associated with trophectoderm-derived cells at specific chromosome regions and genes [71].

The methylation status of the embryonic tissues could favour cell fate transitions that enable further downstream developmental events. In contrast to somatic tissues, which undergo differentiation and acquisition of further methylation, the persistence of a hypomethylated state in extra-embryonic tissues might have favoured the plasticity typical of placenta development in different mammalian organisms, which have evolved different types of placentation with different morphology and different degrees of attachment to the uterus wall [72,73]. How similarity at epigenetic level between cancer cells and trophectoderm is achieved is difficult to explain but could provide important clues about how the functional properties shared between cancer and placenta cells arise.

## 6. Activation of embryonic cell fate transitions in cancer cells

A deeper understanding of the epigenetic and functional similarities between cancer and trophectoderm cells would require the identification of the mechanisms that lead somatic cancer cells to acquire early embryonic features in the context of other elements such as genome instability and hyper-mutagenesis. If RS is one of the early events associated with oncogene activation and loss of tumour suppressors, it is plausible to speculate that RS or RSR plays a role in the acquisition of this trophectoderm-like state in cancer cells. Considering that the acquisition of methylation increases with cell differentiation it is possible that the epigenetic state of cancer cells shared with trophectoderm is linked to de-differentiation events, possibly associated with RS and RSR. This could be compatible with the effect of chemotherapy based on DNA damaging agents, which imposes convergent transcriptional programmes active in therapy-resistant tumours [40]. Alternatively, RS and RSR might favour the emergence of cancer features in somatic cells that have an epigenetic state already more similar to trophectoderm cells. This might be the case for adult stem cells residing in somatic tissues [74].

The alteration of the methylation state might parallel the occurrence of chromatin transitions that lead to the acquisition of a configuration similar to the more open one present in embryonic cells [74]. Intriguingly, recent evidence has shown that RSR in yeast cells leads to a global loss of histones and to chromatin relaxation [75,76]. Furthermore, activation of DNA damage response has been linked to critical ubiquitin-dependent post-translational modifications in linker H1 histones, leading to more relaxed chromatin in the context of DNA double-strand breaks [77]. Intriguingly, consistent with a major switch in chromatin accessibility status occurring in cancer cells, the expression of linker histone H1.0, which is one of the multiple H1 variants, affects the differentiation state of cancer cells and the self-renewal potential of cells that drive tumour growth [3].

Loss of repressive chromatin state might be associated with the reactivation of transposable elements (TEs), including retrotransposons, which can act as promoters, enhancers or insulators, and which are believed to have contributed to the evolution of the placenta through the upregulation of specific gene pathways [78], possibly linked to cancer development. On the one hand, TEs might play a major role in activating innate immune responses based on IFNγ in placental trophectoderm cells, which express high levels of STING [64] and which might contribute to fetus responses to viruses in the absence of a fully active immune system. On the other hand, TEs reactivation in non-placental cells might activate cGAS-STING-dependent inflammatory processes that promote cellular reprogramming that accompanies cellular transformation. Therefore, the transition to an epigenetic configuration present in trophectoderm cells might favour the emergence of properties associated with trophoblast in cancer cell precursors. This could be particularly relevant in cells that have mutated BRCA1 gene, the inactivation of which has been shown to de-repress DNA sequences associated with TEs such as tandemly repeated satellite DNA that can phenocopy *BRCA1* loss in cell cycle checkpoint defects, DNA damage and genomic instability [79].

If the link between RSR and chromatin de-repression is confirmed in other systems, it could be the starting point to understand the molecular mechanisms predisposing to alterations in the epigenetic configuration following the occurrence of RS and activation of RSR. Furthermore, a role for ATR-dependent RSR in driving these changes might explain the requirement for ATR cancer cell survival as reflected by the efficacy of ATR inhibitors in killing tumour cells [80].

Significantly, PD-L1, which is a major protein involved in T-cell suppression in cancer and placenta cells, the targeting of which has shown effective responses in a subset of tumours, has been shown to be upregulated in response to genotoxic stress [81]. This upregulation requires ATM/ATR/Chk1 kinases and is enhanced by depletion repair genes such as BRCA2 through STAT1–3 signalling and IRF1, which are involved in IFNγ-mediated responses triggered by cGAS-STING pathway activated in the presence of RS [81].

Intriguingly, recent machine learning-based methods identified a common root in different types of cancer characterized by the presence of stemness features [82,83]. Significantly, these studies highlighted the presence of a strong link between stemness features and genome instability, especially occurring after mutations in repair genes such as BRCA1/2. In all these cases, stemness was linked to PD-L1 expression [82].

If these factors, which appear to be separate, are considered as a natural progression of events that start with the occurrence of RS, it can be speculated that ATR-dependent RSR contributes to the acquisition of stemness features together with the activation of PD-L1 in the context of more general inflammatory and immunosuppressive responses.

Stemness features might then confer typical cancer properties by supporting the emergence of embryo development pathways linked to placenta formation. In particular, RSR-dependent de-differentiaton to a stem-like state or activation of RSR in stem-like cells might recapitulate the phenomenon by which early embryonic stem cells undergo differentiation towards more proximal lineages [84,85].

Response to stress has been linked to the formation and the evolution of the trophectoderm cells and more in general of the placenta, in which initial inflammatory events driven by contact with the maternal tissues have led to the evolution of anti-inflammatory and anti-maternal immunity strategies, among which trophectoderm expression of PD-L1 plays a central role [9,10].

A key feature of trophoblast is its direct derivation from cells with totipotent capacity. The zygote and the initial stages of embryonic development in mammals are considered totipotent as they can give rise to embryonic and extra-embryonic lineages and, therefore, are capable to promote the formation of the conceptus. The zygote is by definition a cell originated by a stressful event such as the fusion of the male and female germ cells [86]. The totipotent state is characterized by de-repression of a number of genes, including retrogenes associated with the stress response [86]. Similar to the zygote and early embryonic cell stages, it can be speculated that RSR in cancer cells activates the genes linked to the establishment of totipotency. A totipotent-like state in embryonic stem cells that recapitulates the zygotic transcriptional activation is driven by genes such as DUX4, which directs the totipotency programme inducing several gene products, including Zscan4 and many retroelements [87,88]. Significantly, DUX4 is sometimes mutated in cancer cells [89,90]. Zscan4 instead contributes to genome stability of embryonic stem cells and has been shown to be expressed in cancer cells [91]. The overexpression of many genes active in totipotent germ cells has been documented in highly invasive lung cancers [48]. Cancer cells also bear mutations in genes that control the totipotent state, the inactivation of which might lead to the acquisition of totipotent-like features [92]. Among these, there are Tet 1/2/3 [93] and HDACs [94].

Significantly, some types of cancers deriving from totipotent germ cells such as the chorion carcinoma, which is a rare tumour occurring in the context of gestational trophoblastic disease, are among the most invasive human tumours [95]. These cancers are the best example of tumours deriving from totipotent stem cells as they can give rise to several differentiated tissues and can recapitulate aberrant placentation. These tumours derive from the fertilization of a female oocyte that has lost DNA (complete mole) or by the fertilization of an intact germ cell by multiple sperms leading to tri- and tetraploid genotypes (partial moles) [96,97]. The genome configuration in these germ cells with unbalanced ploidy and gene dosage might lead to extreme RS and activation of RSR, the effect of which might divert the zygote towards a placenta-like differentiation programme in which the embryo proper is unable to develop. It can be speculated that some of the molecular processes linked to these events might re-emerge at later stages in adult stem cells conferring the migratory, invasive, pro-angiogenetic and immunomodulatory properties shared between cancer and placenta. Interestingly, early clinical attempts of targeting PD-1 inhibitory signalling with pembrolizumab in drug-resistant gestational trophoblastic disease have documented sustained tumour responses, thus suggesting that circumvention of tumour immune evasion is therapeutically effective also in this clinical scenario [98].

## 7. Conclusion and future directions

Here, we tried to sum up the properties shared by cancer and placental cells and discussed the possible reactivation of these features in adult cancer. We have highlighted several properties of cancer cells that may arise from the activation of processes central to placenta development, among which the activation of shared immunoediting mechanisms could play a major role in cancer development. Additional considerations can be made by observing the occurrence and the frequency of cancer in different species.

If malignant cancer recapitulates features of invasive placentation, species in which placentation has not evolved should experience cancer at a different level. In these animals, cancer should be less frequent and/or show milder features, which might not include widespread metastatic diffusion and immune evasion. Several studies have shown that cancer is present at different rates in different species and that some species are intrinsically resistant to malignant cancer [99,100]. Although a higher number of cells and/or a higher number of cell doublings should increase the chance for mutations to occur and accumulate, some animals of large size are protected from cancer. This is known as Peto's paradox and it might be explained by the acquisition of resistance to cancer through natural selection [99]. Different mechanisms that protect and promote cancer-free status have been discovered in large or long-lived animals. For example, elephants have at least 20 copies of the tumour-suppressor gene p53 [101], whereas in naked mole-rats (*Heterocephalus glaber*) high-molecular-mass hyaluronan could mediate cancer resistance [102]. Therefore, natural selection and likely reproductive rates, predation, lifespan and many other factors determine overall cancer rates. It is, however, worth mentioning that placental mammals, in particular the ones with highly invasive haemochorial placentas, have the highest malignant cancer rates among all animals [103]. It would be interesting to compare different types of placentation with the rates of malignancies in different species. Interestingly, metastatic solid tumours have not been reported so far in non-placental mammals such as monotremes, which lay eggs rather than bearing live young [103]. Intriguingly, non-mammalian organisms, which rely more on innate immunity-based mechanisms for their defence from microbes and parasites, suffer from tumours that have different behaviours from those occurring in mammals. Indeed, tumours in fish mostly affect blood cells, and solid tumours including carcinomas and sarcomas which are rare in these species, are mostly locally invasive and rarely metastatic [104]. Amphibians are also very resistant to cancer development as only two types of tumours are known in these species, including the renal adenocarcinoma of *Rana pipiens* and the lymphosarcoma of *Xenopus laevis* [105]. Although the remarkably low frequency of metastatic features of cancers occurring in non-mammalian organisms might be due to species-specific cancer-resistance mechanisms, it strongly correlates with lack of placentation.

The relationship between invasive placentation and metastasis could be explained by antagonistic pleiotropy [15]. According to this hypothesis, metastatic cancer could be a negative cost associated with the evolution of invasive placentation. Basically, the evolution of placentation might have greatly increased the fitness of the organism in which it evolved and therefore it might have been positively selected. In this case, the consequence of bearing a genetic programme predisposing to metastatic behaviour, which occurs later in life, would have been tolerated and not counter-selected. However, it could also be argued that a link between placentation and species-specific malignancy rates is more related to mechanisms that suppress invasion and that evolved in the organisms in which placentation appeared [15]. In this model, which involves positive pleiotropy, maternal responses to embryo attachment might have led to the loss of invasive placentation in some lineages of mammals, suppressing trophoblast invasion. These mechanisms might have played a role in protecting organisms against cancer invasiveness. These observations might explain the different relations among organisms with non-invasive placentation and metastatic cancers, including the ones observed in marsupials, in which metastatic cancer occurs despite the lack of invasive placentation.

However, we would like to point out that the degree of invasiveness might not be the major factor correlating with metastatic cancer. Invasiveness appeared before placentation during animal evolution as it is active in wound healing and EMT. Therefore, placenta invasiveness might not directly correlate with some of the malignant cancer features although it can increase cancer aggressiveness in some species. It is instead more likely that evolution of immunotolerance mechanisms towards fetal neo-antigens, especially the ones based on sophisticated immune controls orchestrated by T-reg cells at the feto-maternal immune barrier, might have played a more important role in the acquisition of mechanisms by which cancer cells escape immune control and invade surrounding tissues.

Overall, these observations might help to define a novel area of cancer research, which could take advantage of the comparisons between cancer rates and developmental strategies of different species. In particular, the study of mammalian placentation could help to identify novel anti-tumour targets able to suppress the immune tolerance shared between placentation and cancer. It is indeed likely that placentation has evolved a number of yet to be known strategies to promote immunotolerance. Their discovery could allow the identification of new targets for cancer therapy.

The study of placenta-specific genes re-expressed in cancer cells might also give the possibility of identifying new targets of therapy with limited toxicity. Among these, an example worth mentioning are PLAC1 and syncytins, which are placenta-specific genes [11]. PLAC1 is re-expressed in prostate, breast and ovary cancer and is a potential target for antibody–drug conjugate-based prostate cancer immunotherapy [106]. Syncytins, a family of placenta-specific genes evolved from retroelements and involved in placenta cell fusion are also highly expressed in breast and other cancers, where they contribute to cancer cell fusion with endothelium, a process that can be targeted by syncytin inhibiting peptides [107].

To conclude, if cancer progression and placenta formation share common molecular pathways that are physiologically required only for pregnancy, they might be the perfect target for cancer therapy with relatively low toxicity.

## Data accessibility

This article has no additional data.
